# Effects of tree species and topography on soil and microbial biomass stoichiometry in Funiu Mountain, China

**DOI:** 10.1186/s12898-020-00332-4

**Published:** 2020-12-09

**Authors:** Yaowu Tian, Dong Qiao, Shaojun Xu, Ning Wang

**Affiliations:** Forestry College, Henan University of Science & Technology, No. 263 Kaiyuan Avenue, Luoyang, 471000 China

**Keywords:** Ecological stoichiometry, Soil microbes, Tree species, Topography, Homeostasis

## Abstract

**Background:**

Soil and microbial biomass stoichiometry plays an important role in understanding nutrient cycling in terrestrial ecosystems. However, studies on soil and microbial biomass stoichiometry in forests are rare. This study investigated the effect of tree species and topographic factors on the ecological stoichiometry of soil and soil microbial biomass.

**Methods:**

Three types of forest stands (*Quercus variabilis*, *Larix principis*-*ruprechtii*, and *Cotinus coggygria* Scop.) in the Beiru River basin of Funiu Mountain were analyzed in September 2018. Six slope positions (sunny bottom slope, sunny middle slope, sunny top slope, shady bottom slope, shady middle slope, and shady top slope) were selected, and the total number of sampling plots was 108. The stoichiometric indices of soil and microbial biomass were determined.

**Results:**

At a depth of 0–10 cm, the soil organic C contents in different stands followed the order of *C. coggygria* (27.7 ± 5.2 g/kg) > *Q. variabilis* (24.5 ± 4.9 g/kg) > *L. principis*-*ruprechtii* (20.8 ± 4.3 g/kg) (P < 0.05). The soil organic C contents at depths of 0–10 cm with different slope aspects and at different slope positions also showed significant differences (P < 0.05). The highest MBC content was observed at the slope bottom (1002 ± 157 mg/kg), whereas the lowest was observed at the slope top (641 ± 98.3 mg/kg). Redundancy analysis showed that the contribution of tree species to these differences was 57.1%, whereas that of topographical factors was 36.2%.

**Conclusions:**

Tree species more significantly affected soil nutrients and microbial biomass C, N and P than did topographic factors.

## Introduction

Soil microbes mineralize C, N and P and other nutrient elements in the soil through decomposition and release them into the soil, promoting the material cycle of the ecosystem [1, 2]. Soil microbial community structure and functional characteristics influence the biogeochemical cycle process [3, 4]. Ecological chemometry is a science that studies the balance of C, N, P and other elements in ecosystem interactions and processes [5]. Cleveland and Liptzin [6] integrated the soil microbial biomass data of the global terrestrial ecosystem and thought that the proportions of C, N and P in the soil microbes was similar to the “Redfield ratio”. The soil microbes also display homeostasis. The soil microbial biomass N:P can be used as a tool to assess nutrient limitation [7, 8].

The C, N and P stoichiometric ratios of the soil microbial biomass can determine the nutritional status and restrictions on microbial growth [7, 9]. Ren et al. [10] studied a vegetation restoration area in the Loess Plateau, China, and found that nutrient limitation could be determined by soil microbial biomass N:P. In addition, plants display homeostasis, and a “Redfield-like ratio” is present in plants; as influenced by climate change and the biogeographic gradient of soil matrix age, the N:P ratio in plant leaves gradually increases from areas of high latitude to areas of low latitude [11]. The above studies provide new ideas and research methods for the study of nutrient cycling and limitations in forest ecosystems.

Rapid forest vegetation growth consumes excessive soil nutrients [12–14]. Stand growth is gradually constrained by soil nutrients, especially by soil N and P. However, forest tree species, slope aspect and position and other conditions are not always consistent. The soil in the bottom slope and that in the shady slope often have better moisture and soil conditions, and the difference in soil nutrients further contributes to the variation in the N:P in tree leaves [15–17].

Soil microbial biomass is a critical factor in ecosystem nutrient cycling [16–18], and knowledge about its relation with the ecological stoichiometry of soil is crucial for us to gain insight into the nutrient cycling of terrestrial ecosystems [19–22]. However, there is currently little literature available regarding the variation in the ecological stoichiometry of soil microbes at different forest soil depths. Meanwhile, differences in soil nutrient content, bulk density and moisture content between different soil depths often cause variations in soil microbial biomass at different soil depths [23–28]. Little is understood about the effect of tree species and topography on the ecological stoichiometry of soil and soil microbial biomass.

Based on this information, this paper took Funiu Mountain, China, as the research site to study the effects of tree species and topography on soil and microbial biomass stoichiometry. The study aimed to test the following hypotheses: (1) Environmental factors (tree species, slope position and aspect) affect the C, N and P contents in the soil and microbial biomass of different soil depths (0–10 cm, 10–20 cm) and their ecological stoichiometry. (2) Forest soil microbes display homeostasis. (3) Nutrient limitations of the forest ecosystem in the study area can be assessed using the soil microbial biomass N:P.

## Materials and methods

### Study area

The study area, with an elevation of 780–1270 m, is located in the upper reaches of the Beiru River in Funiu Mountain, China. It has a warm temperate continental monsoon climate, with an average annual temperature of 12.4 **°**C, an annual sun exposure duration of 2103 h, a frost-free period of 198 days, and an average annual precipitation of 670 mm. The soil type is mainly brown soil, according to the soil classification criteria enacted by the General Administration of Quality Supervision, Inspection and Quarantine of the People’s Republic of China [29]. The main tree species are secondary deciduous broadleaf trees, such as *Quercus variabilis, Cotinus coggygria* Scop. and *Acer mono* Maxim. In the 1950s, large areas of *Larix principis*-*ruprechtii*, *Pinus tabuliformis* Carr., and other coniferous trees were planted on Funiu Mountain, China. The herbaceous plants mainly include *Miscanthus*, *Cyperus rotundus* L., *Thalictrum aquilegifolium*, *Setaria viridis* (L.) Beauv., and *Spiraea cantoniensis* Lour., and the forest coverage percentage is 83.5% in the study area [30].

### Soil sampling

In September 2018, the three most representative forest stands in the investigated area, i.e., *Q. variabilis*, *L. principis*-*ruprechtii* and *C. coggygria* Scop., were selected. The stands were basically consistent in appearance, i.e., tree and crown densities. Three typical shady slopes and three sunny slopes were selected from each forest stand (the definitions for shady slopes and sunny slopes were based on the descriptions in GB 26424-2010-T Technical Specifications for Forest Resources Planning, Design and Investigation of China). Three typical non-catena plots (10 m × 10 m) were set up on each slope from bottom to top according to the elevation, i.e., bottom slope, middle slope and top slope. There were 108 (3 × 3×2 × 2×3) sample plots in total (including three replicates for each combination). In each sample plot, 5–6 sampling points were randomly selected, with a distance between sampling points of about 10 m. The floating leaves were gently removed, and soil samples were collected from depths of 0–10 and 10–20 cm with a soil auger whose bit length was 30 cm. The soil samples from the same depth of each sample plot were fully mixed. One kg of the above soil sample was taken back to the laboratory and screened through a 2-mm sieve to remove materials such as plant roots, stones, and litter. One part of fresh soil was preserved at 4 °C and the contents of soil microbial biomass C, N and P were measured within 10 days. The remaining soil samples were dried naturally and then screened through a 20-mesh sieve to obtain the 1-mm soil sample and then screened through a 0.149-mm sieve to measure the total nutrients of the soil. The specific information about the sampling points is shown in Table 1.

**Table 1:** Basic information of the sample plots

Tree species	Sample plot	Slope position	Longitude	Latitude	Altitude (m)	Aspect	Gradient (°)	Litter thickness (cm)
*Q. variabilis*	1	Bottom slope (VS1)	112° 12′ 44.46″	33° 44′ 55.23″	1209	Sunny slope	25	3.4
	2	Middle slope (MS1)	112° 12′ 56.39″	33° 44′ 54.19″	1307	Sunny slope	24	2.7
	3	Top slope (RS1)	112° 13′ 6.61″	33° 44′ 57.59″	1360	Sunny slope	32	2.2
	4	Bottom slope (VS2)	112° 12′ 32.38″	33° 45′ 28.02″	1086	Shady slope	31	4.6
	5	Middle slope (MS2)	112° 12′ 44.69″	33° 45′ 27.57″	1184	Shady slope	28	4.7
	6	Top slope (RS2)	112° 12′ 58.48″	33° 45′ 27.81″	1240	Shady slope	30	3.2
	7	Bottom slope (VS1)	112° 13′ 11.39ʺ	33° 44′ 20.81ʺ	1287	Sunny slope	34	4.3
	8	Middle slope (MS1)	112° 13ʹ 15.30ʺ	33° 44′ 26.45ʺ	1355	Sunny slope	31	3.5
	9	Top slope (RS1)	112° 13ʹ 19.03ʺ	33° 44ʹ 32.04ʺ	1440	Sunny slope	25	3.1
	10	Bottom slope (VS2)	112° 13ʹ 12.07ʺ	33° 44ʹ 40.88ʺ	1370	Shady slope	27	4.1
	11	Middle slope (MS2)	112° 13ʹ 18.55ʺ	33° 44ʹ 37.46ʺ	1421	Shady slope	28	4.0
	12	Top slope (RS2)	112° 13ʹ26.91ʺ	33° 44ʹ34.64ʺ	1467	Shady slope	23	3.1
	13	Bottom slope (VS1)	112° 12ʹ 32.32″	33° 44ʹ 22.89″	1149	Sunny slope	25	3.5
	14	Middle slope (MS1)	112° 12ʹ 36.69″	33° 44ʹ 26.32″	1173	Sunny slope	28	3.8
	15	Top slope (RS1)	112° 12ʹ38.10″	33° 44ʹ31.79″	1243	Sunny slope	29	3.5
	16	Bottom slope (VS2)	112° 12ʹ37.97″	33° 44ʹ46.66″	1136	Shady slope	25	5.7
	17	Middle slope (MS2)	112° 12ʹ 39.44″	33° 44ʹ 40.48″	1205	Shady slope	21	4.3
	18	Top slope (RS2)	112° 12ʹ 42.21″	33° 44ʹ 34.73″	1256	Shady slope	23	4.2
*L. principis*-*ruprechtii*	19	Bottom slope (VS1)	112° 12′ 42.65″	33° 45′ 39.63″	1069	Sunny slope	24	5.3
	20	Middle slope (MS1)	112° 12′ 52.72″	33° 45′ 37.37″	1195	Sunny slope	25	4.6
	21	Top slope (RS1)	112° 13′ 3.13″	33° 45′ 35.62″	1311	Sunny slope	26	3.8
	22	Bottom slope (VS2)	112° 12′ 47.91″	33° 45′ 43.40″	1153	Shady slope	20	4.5
	33	Middle slope (MS2)	112° 12′ 57.87″	33° 45′ 41.23″	1226	Shady slope	34	4.2
	24	Top slope (RS2)	112° 13′ 5.34″	33° 45′ 38.53″	1331	Shady slope	25	3.2
	25	Bottom slope (VS1)	112° 12ʹ7.23″	33° 44ʹ 54.27″	1109	Sunny slope	29	4.3
	26	Middle slope (MS1)	112° 11ʹ 57.30″	33° 44ʹ 59.39″	1212	Sunny slope	25	3.8
	27	Top slope (RS1)	112° 11ʹ47.41″	33° 45ʹ0.30″	1345	Sunny slope	34	2.5
	28	Bottom slope (VS2)	112° 12ʹ13.68″	33° 45ʹ10.37″	1093	Shady slope	27	4.3
	29	Middle slope (MS2)	112° 12ʹ4.40″	33° 45ʹ8.13″	1163	Shady slope	30	4.5
	30	Top slope (RS1)	112° 11ʹ52.01″	33° 45ʹ4.55″	1301	Shady slope	26	3.2
	31	Bottom slope (VS1)	112° 14ʹ14.85″	33° 45ʹ55.27″	1078	Sunny slope	21	5.6
	32	Middle slope (MS1)	112° 14ʹ24.14″	33° 45ʹ50.39″	1200	Sunny slope	31	4.4
	33	Top slope (RS1)	112° 14ʹ37.06″	33° 45ʹ47.30″	1388	Sunny slope	27	5.1
	34	Bottom slope (VS2)	112° 14ʹ40.09″	33° 46ʹ8.33″	1087	Shady slope	21	4.5
	35	Middle slope (MS2)	112° 14ʹ40.08	33° 45ʹ59.66″	1202	Shady slope	25	4.2
	36	Top slope (RS2)	112° 14ʹ42.64	33° 45ʹ49.76″	1388	Shady slope	25	2.6
*C. coggygria* Scop.	37	Bottom slope (VS1)	112° 12′ 27.81″	33° 46′ 3.39″	993	Sunny slope	28	5.5
	38	Middle slope (MS1)	112° 12′ 36.44″	33° 46′ 2.66″	1048	Sunny slope	34	6.6
	39	Top slope (RS1)	112° 12′ 43.91″	33° 46′ 4.56″	1123	Sunny slope	35	6.2
	40	Bottom slope (VS1)	112° 12′ 37.68″	33° 45′ 52.39″	1044	Shady slope	25	5.8
	41	Middle slope (MS2)	112° 12′ 45.60″	33° 45′ 55.53″	1141	Shady slope	26	5.6
	42	Top slope (RS2)	112° 12′ 54.37″	33° 45′ 55.53″	1204	Shady slope	27	4.6
	43	Bottom slope (VS1)	112° 11′ 50.03	33° 43′ 16.02″	1408	Sunny slope	24	5.6
	44	Middle slope (MS1)	112° 11′ 47.12	33° 43′ 22.11″	1448	Sunny slope	28	7.2
	45	Top slope (RS1)	112° 11′ 46.77	33° 43′ 30.00″	1535	Sunny slope	35	3.5
	46	Bottom slope (VS1)	112° 11′ 39.22	33° 43′ 50.93″	1414	Shady slope	34	5.6
	47	Middle slope (MS2)	112° 11′ 39.96	33° 43′ 44.16″	1483	Shady slope	25	5.4
	48	Top slope (RS2)	112° 11′ 40.80	33° 43′ 35.35″	1530	Shady slope	26	6.2
	49	Bottom slope (VS1)	112° 12′ 15.96	33° 45′ 44.48″	1005	Sunny slope	28	6.5
	50	Middle slope (MS1)	112° 12′ 9.66	33° 45′ 40.66″	1066	Sunny slope	27	5.4
	51	Top slope (RS1)	112° 12′ 0.29	33° 45′ 37.30″	1166	Sunny slope	31	4.5
	52	Bottom slope (VS2)	112° 12′ 21.07	33° 45′ 31.34″	989	Shady slope	32	7.8
	53	Middle slope (MS2)	112° 12′ 17.06	33° 45′ 33.99″	1018	Shady slope	28	7.5
	54	Top slope (RS2)	112° 12′ 13.97	33° 45′ 37.13″	1068	Shady slope	24	6.4

The procedures of this study were approved by the Environmental Protection Bureau of Lushan County, Henan.

### Measurement indices

The proposed method is based on the complexation of malachite green with phosphomolybdate under acidic conditions.

The total C and N in the soil were determined using a Vario Max C&N analyzer (Elemental Ltd., UK). The contents of C, N and P in soil microbial biomass were determined using chloroform fumigation extraction (CFE) [7, 31, 32]. Each soil sample was made up of subsamples marked “fumigated” and “unfumigated”. Before fumigation, soil samples were cultured at 4 **°**C for 7 days. The soil samples (equivalent to 3 g in dry weight) were fumigated with ethanol-free chloroform at 25 °C for 24 h, and then C and N were extracted using 0.5-M K_2_SO_4_. P was extracted using 0.5M NaHCO_3_ (pH 8.5) [33]. The unfumigated soil was extracted in the same way, and the extract was filtered through a 0.45-μm syringe-driven filter. Soil organic carbon (SOC) and total nitrogen were measured using a total organic carbon analyzer (Shimadzu TOC-V CPH, Shimadzu Corp., Kyoto, Japan). The transforming factors of microbial biomass C and N are 0.45 and 0.54, respectively [31, 32, 34]. MBC and MBN represent the concentration differences between the fumigated and unfumigated samples [35]. The extractant of P was transferred to a 96 microwell plate, and the content of P was measured by the ammonium molybdate-malachite green method [36]. The transforming factor of microbial biomass P used in this paper was 0.40 [31, 35].

### Statistical analysis

The mass ratio is often used in terrestrial ecosystem research [37], while the molar ratio is often used in aquatic ecology research; thus, the former was adopted for the stoichiometric ratios of C, N and P of soil and microbial biomass. Microsoft Excel 2010 was used for experimental data sorting and plotting. SPSS 20.0 was used for statistical analysis. Levene’s test was performed to determine the homogeneity of variance. For data with heterogeneous variance, logarithmic transformation was performed. For data with a normal distribution, one-way ANOVA was used for comparisons among groups (α = 0.05). Otherwise, nonparametric Kruskal–Wallis one-factor ANOVA was used. The p values were adjusted by the false discovery rate. Redundancy analysis was performed to determine the contributions of environmental factors to stoichiometry using CANOCO 5 to visually exhibit the relationship between variables and response variables [38]. A general linear model (GLM) was used to assess the effects of tree species, slope aspect, position and their combined effect. Pearson correlation analysis was used to analyze the relationship between the contents of C, N and P in soil and soil microbial biomass and the corresponding C:N, C:P and N:P ratios.

SMATR2.0 [39] was used for the standardized major axis (SMA) analysis of the relationship between the C, N and P contents in soil and those in soil microbial biomass [6, 31] to explore the homeostasis of the soil microbial biomass. SMA analysis presents the “best” binary fitting line between two variables. The relationship between the C, N, and P contents in soil and those in soil microbial biomass was expressed by the equation lgy = *a*+*b*lgx, where *a* is the intercept and b is the slope. When the slope *b* is not significantly greater than 1, the relationship between the two variables is isometric [6].

## Results

### Effects of tree species and topography on the C, N and P contents

**Fig. 1:**
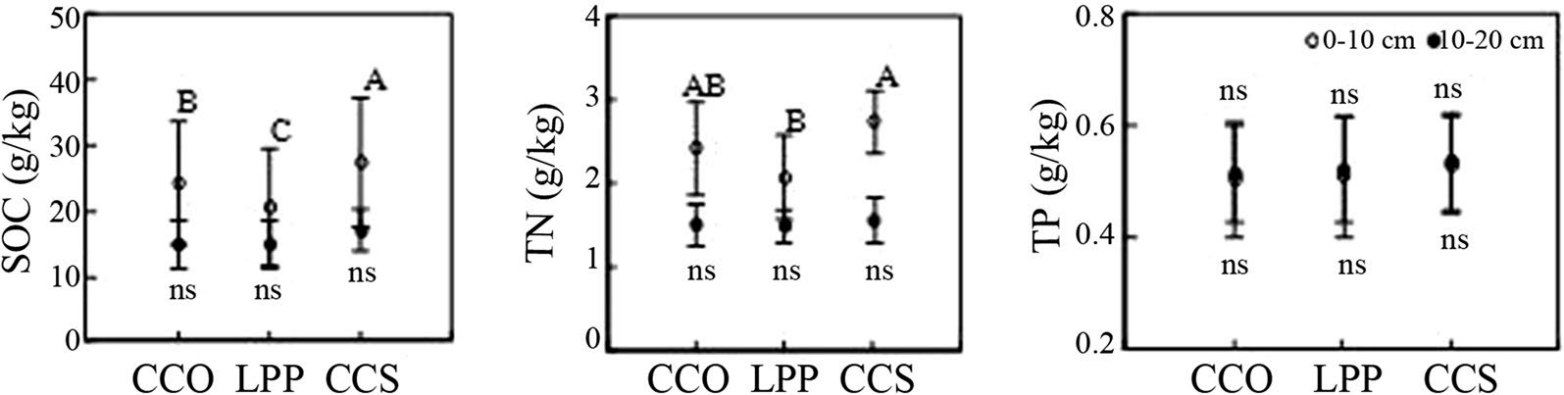
Effect of tree species on the distribution of soil C, N and P contents. A different capital letter or letter combination indicates a significant difference in the SOC content among tree species. CCO: *Q. variabilis*; LPP: *L. principis*-*ruprechtii*; CCS: *C. coggygria* Scop.; ns: not significant

At depths of 0–10 cm, the SOC contents of the soil of different tree species was in the order of *C. coggygria* Scop. (27.7 ± 9.8 g/kg) > *Q. variabilis* (24.5 ± 9.1 g/kg) > *L. principis*-*ruprechtii* (20.8 ± 9.8 g/kg) (p < 0.05; Fig. 1). There was no significant difference in the SOC content at 10–20 cm. The SOC content significantly differed (p < 0.05) at different soil depths of the three tree species. The effect of tree species on soil total nitrogen (TN) content was similar to that of SOC, and its effect on soil total phosphorus (TP) content was not significant. The variation in TP content was not significant in relation to tree species and soil depth.

**Fig. 2:**
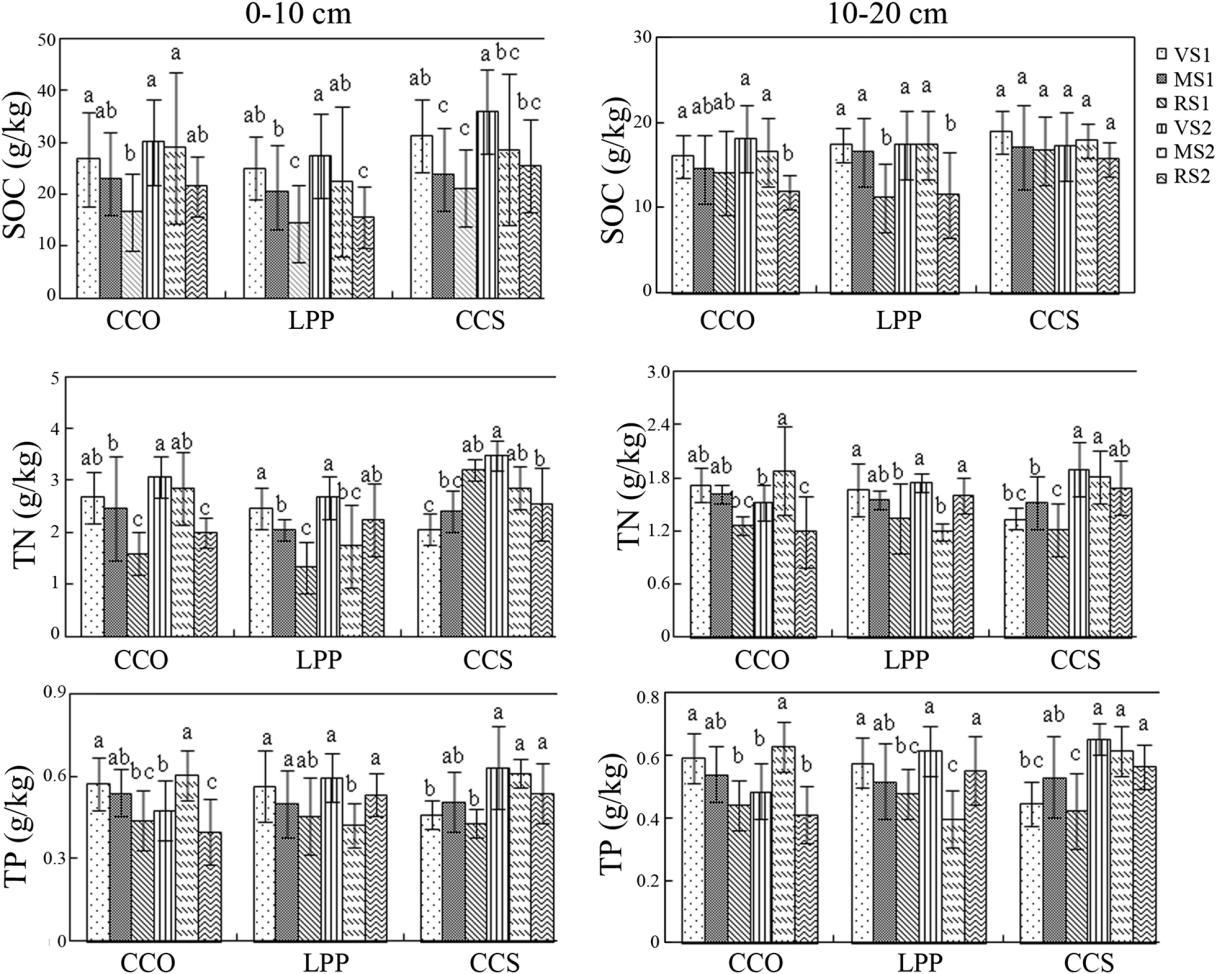
Effects of geographic factors and tree species on the soil C, N and P contents. The difference was significant between slope positions of different tree species represented by different small letters (p < 0.05). CCO: *Q. variabilis*; LPP: *L. principis*-*ruprechtii*; CCS: *C. coggygria* Scop.; VS1: sunny bottom slope; MS1: sunny middle slope; RS1: sunny top slope; VS2: shady bottom slope; MS2: shady middle slope; RS2: shady top slope

At 0-10 cm, the SOC contents of *Q. variabilis*, *L. principis*-*ruprechtii* and *C. coggygria* Scop. were in the following order: bottom slope > middle slope > top slope, with the highest value in shady bottom slope and the lowest value in sunny top slope (Fig. 2). The difference between the shady/sunny bottom slope and sunny top slope was significant, and the SOC content in the shady slope was higher than that in the sunny slope overall. At 10–20 cm, the SOC content was greater in the bottom slope than in the top slope. However, there was no significant difference between different slope positions for the sunny slope of *Q. variabilis* and the shady/sunny slope of *C. coggygria* Scop. At 0–10 cm, the soil TN contents in the shady and sunny slopes of *Q. variabilis*, the sunny slope of *L. principis*-*ruprechtii*, and the shady slope of *C. coggygria* Scop. exhibited a similar tendency as that of SOC, that is, the highest content was found in the bottom slope (2.67 g/kg, 2.46 g/kg and 2.05 g/kg, respectively) and the lowest content was found in the top slope (1.6 g/kg, 1.33 g/kg and 1.74 g/kg, respectively), and the difference was significant. There was a significant difference in the soil TN contents between different slope positions for the shady slope of *L. principis*-*ruprechtii* and the sunny slope of *C. coggygria* Scop. However, the variation was not obvious. At 10–20 cm, the soil TN contents of the sunny slopes of *Q. variabilis* and *L. principis*-*ruprechtii* and the shady slope of *C. coggygria* Scop. were in the following order: bottom slope > middle slope > top slope, and the difference was significant. However, the other slopes did not show regular variation. Overall, the soil C, N and P contents at 1–10 cm were more noticeably influenced by tree species and topography than those at 10–20 cm.

At 0–10 cm, the TP contents of the sunny slopes of *Q. variabilis* and *L. principis*-*ruprechtii* and the shady slope of *C. coggygria* Scop. were in the following order: bottom slope > middle slope > top slope. However, the difference was not significant (P > 0.05). However, there was no such regular variation in other slopes except that there was a significant difference in the soil TP contents between the shady slope of *Q. variabilis* and that of *L. principis*-*ruprechtii*. At 10–20 cm, the soil TP contents in the sunny slopes of *Q. variabilis* and *L. principis*-*ruprechtii* and the shady slope of *C. coggygria* Scop. were in the following order: bottom slope > middle slope > top slope. There was a significant difference in the TP content between different slope positions for the sunny slope of *Q. variabilis* and that of *L. principis*-*ruprechtii*, whereas there was no significant difference in the TP content between different slope positions for the shady slope of *C. coggygria* Scop. There was no obvious regularity for the other slopes. However, there was a significant difference in the soil TP content.

**Fig. 3:**
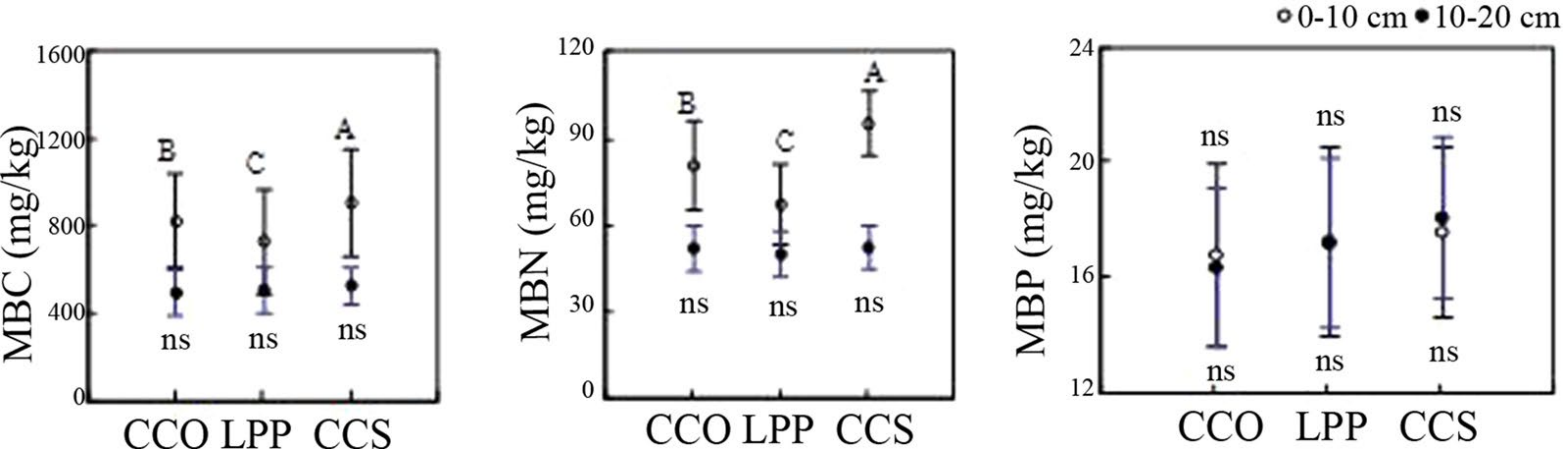
Effect of three tree species on the soil organic C, N and P contents. The vertical line indicates the standard error. Capital letters and lowercase letters represent a significant difference between different tree species at depths of 0–10 cm and 10–20 cm, respectively. According to the Duncan test, different letters indicate a significant difference between tree species (p < 0.05), while “ns” indicates no significant difference

As shown in Fig. 3, at 0–10 cm, the soil microbial biomass C content of different tree species was in the order of *C. coggygria* Scop. > *Q. variabilis* > *L. principis*-*ruprechtii*, with a significant difference between the three (p < 0.05). At 10–20 cm, there was no significant difference in the soil microbial biomass C contents of the three tree species (p > 0.05). At 0–10 cm, the soil microbial biomass C contents of the three tree species were significantly greater than those at 10–20 cm. The soil microbial biomass N content was similar to the C content. However, there were no significant differences in microbial biomass P between the two soil depths, among the three tree species, or in their interactions.

**Fig. 4:**
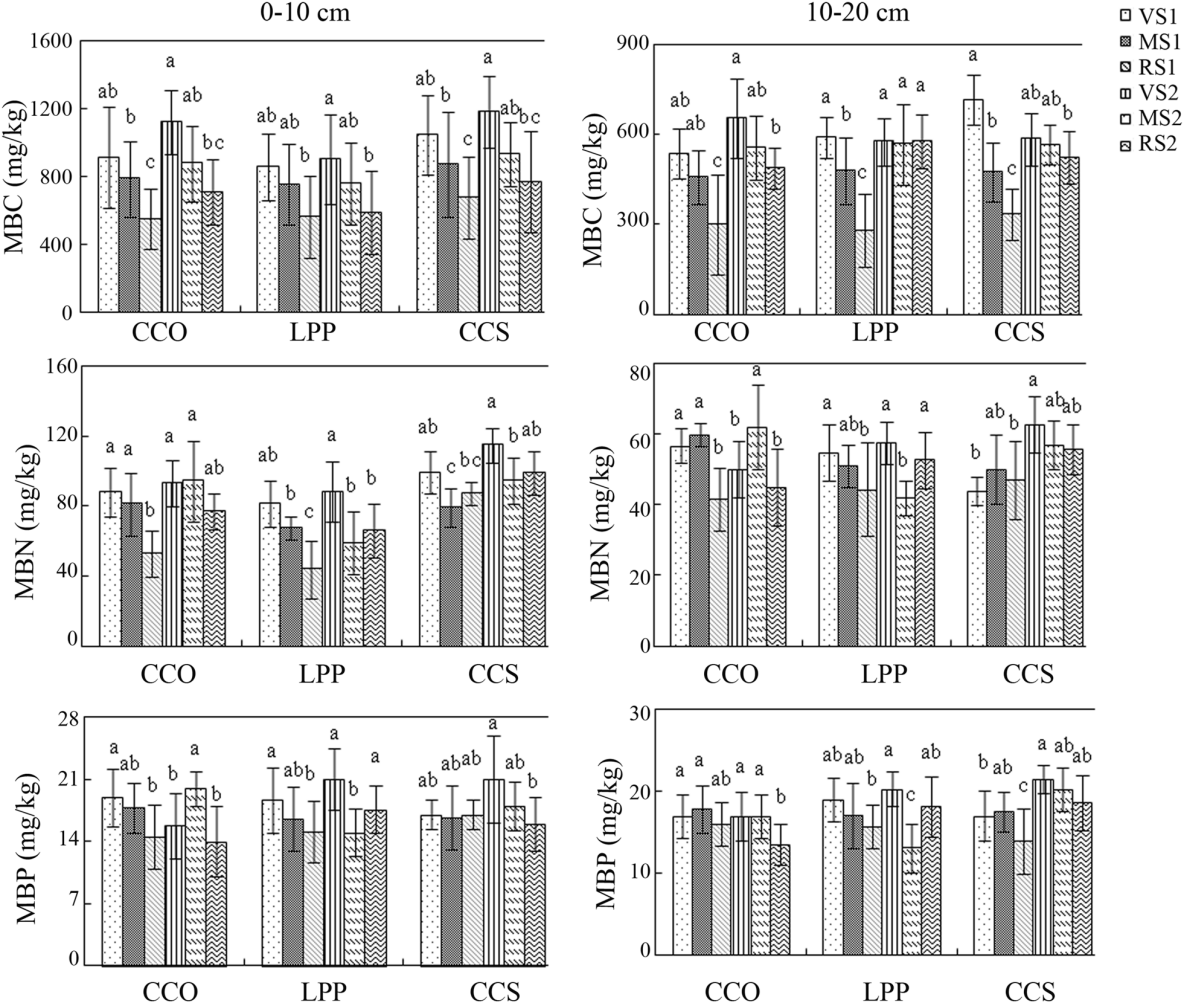
Distribution characteristics of soil MBC, MBN and MBP for different tree species. MBC: microbial biomass C; MBN: microbial biomass N; MBP: microbial biomass P

As shown in Fig. 4, at 0–10 cm, the soil microbial biomass carbon (MBC) contents of *Q. variabilis*, *L. principis*-*ruprechtii* and *C. coggygria* Scop. were invariably highest in the shady bottom slope (1002 ± 157 mg/kg) and lowest in the sunny top slope (641 ± 98.3 mg/kg). At 10–20 cm, the MBC content was invariably highest in the bottom slope and lowest in the top slope, with the exceptions of the shady slopes of *L. principis*-*ruprechtii*. Overall, the soil MBC content in the shady slope was higher than that in the sunny slope.

At 0–10 cm, the changes in the soil microbial biomass nitrogen (MBN) contents of the three tree species did not show regular patterns. The soil MBN content was highest in the shady bottom slope of *C. coggygria* Scop. and lowest in the sunny top slope of *L. principis*-*ruprechtii*. At 10–20 cm, the MBN content in the sunny slope of *L. principis*-*ruprechtii* and shady slope of *C. coggygria* Scop. was in the order of bottom slope > middle slope > top slope. There was no significant difference in the soil MBN between the shady and sunny slopes of the three tree species.

At 0–10 cm, the soil microbial biomass phosphorus (MBP) contents of *Q. variabilis*, *L. principis*-*ruprechtii* and *C. coggygria* Scop. did not show regular changes. Its contents in the sunny slope of *Q. variabilis*, sunny slope of *L. principis*-*ruprechtii*, and shady slope of *C. coggygria* Scop. were in the order of bottom slope > middle slope > top slope, and the difference was significant. The highest content of soil MBP was in the bottom slope of *C. coggygria* Scop (19.0 mg/kg), and the lowest was in the shady top slope of *Q. variabilis* (16.5 mg/kg). At 10–20 cm, no significant differences in the soil MBP contents were observed among the three slope positions of the sunny slopes of *Q. variabilis*, *L. principis*-*ruprechtii* and *C. coggygria* Scop.

### Effects of tree species and topography on soil and microbial biomass stoichiometry

As shown in Tables 2 and 3, the C:N ratio in *Q. variabilis* soil was highest on the sunny bottom slope, and the soil C:N ratio was significantly different between the sunny bottom slope and the other slope positions. The C:N ratio in *C. coggygria* Scop. soil was highest in the sunny bottom slope, and there was no significant difference in the C:N ratio between the sunny bottom slope and the other slope positions. The C:N ratio in the *L. principis*-*ruprechtii* soil was highest in the shady top slope, and there was no difference between the different slope positions of *L. principis*-*ruprechtii*. The trends of soil C:P and N:P were basically consistent with that of soil C:N.

**Table 2:** Stoichiometric characteristics of soil and microbial biomass

Tree species	Slope position	C:N		C:P		N:P		MBC:MBN		MBC:MBP		MBN:MBP	
		0–10 cm	10–20 cm	0–10 cm	10–20 cm	0–10 cm	10–20 cm	0–10 cm	10–20 cm	0–10 cm	10–20 cm	0–10 cm	10–20 cm
*Q. variabilis*	Sunny bottom slope (VS1)	16.3 ± 1.6a	13.2 ± 0.9a	34.5 ± 3.32a	30.2 ± 4.5a	3.3 ± 0.6a	3.1 ± 0.8a	3.9 ± 0.5a	4.5 ± 0.6a	34.6 ± 11.6a	31.6 ± 3.6a	10.2 ± 1.1a	9.7 ± 2.45a
	Sunny middle slope (MS1)	13.4 ± 1.4b	11.4 ± 1.23a	33.1 ± 8.5a	26.3 ± 2.56a	2.8 ± 0.2a	2.3 ± 0.7b	3.7 ± 0.3a	3.5 ± 0.1b	31.9 ± 3.7a	22.6 ± 3.3b	9.1 ± 0.71a	7.6 ± 1.2b
	Sunny top slope (RS1)	10.3 ± 1.4c	9.5 ± 0.4b	26.7 ± 11.6b	22.9 ± 11.2b	2.2 ± 1.1b	2.1 ± 1.1b	3.5 ± 0.7ab	3.8 ± 0.1c	26.7 ± 6.19b	21.7 ± 6.7b	7.7 ± 1.4b	7.4 ± 2.6b
	Shady bottom slope (VS1)	12.8 ± 1.1b	12.4 ± 1.4a	33.8 ± 8.9a	26.1 ± 9.0a	2.7 ± 1.8a	2.5 ± 0.6b	3.5 ± 0.6ab	4.2 ± 0.6a	31.2 ± 8.8a	28.5 ± 7.4a	8.7 ± 1.5ab	7.5 ± 3.4b
	Shady middle slope (MS2)	11.5 ± 1.6bc	11.8 ± 2.2ab	30.6 ± 11.1a	23.1 ± 1.5b	2.5 ± 1.3ab	1.8 ± 0.3bc	3.3 ± 0.5ab	3.6 ± 0.2b	30.5 ± 6.8a	25.5 ± 6.1b	8.2 ± 1.7b	5.8 ± 2.5c
	Shady top slope (RS2)	11.4 ± 1.5bc	9.7 ± 1.3b	24.67 ± 10.9b	22.6 ± 7.6b	1.9 ± 0.9b	1.4 ± 0.6c	3.1 ± 1.4b	3.5 ± 0.4b	31.4 ± 5.7a	31.1 ± 11.6a	9.4 ± 0.6a	9.1 ± 1.7a
*L. principis*-*ruprechtii*	Sunny bottom slope (VS1)	8.9 ± 1.09b	9.21 ± 1.1a	9.6 ± 1.7a	9.4 ± 0.9a	1.07 ± 0.1b	1.1 ± 0.03a	4.1 ± 1.4a	6.5 ± 1.8a	56.1 ± 4.1a	63.9 ± 8.6a	11.3 ± 1.8a	11.5 ± 1.4a
	Sunny middle slope (MS1)	9.7 ± 0.7ab	9.5 ± 0.6a	9.8 ± 1.6a	9.9 ± 1.1a	1.3 ± 0.07a	0.8 ± 0.06b	3.9 ± 0.7b	4.6 ± 1.3c	51.2 ± 14.1b	65.5 ± 18.7a	9.5 ± 2.1b	9.8 ± 6.1ab
	Sunny top slope (RS1)	10.9 ± 0.8a	9.8 ± 0.3a	10.5 ± 1.3a	10.2 ± 1.2a	1.4 ± 0.15a	1.2 ± 0.1a	3.7 ± 0.8b	5.2 ± 1.6b	42.4 ± 17.5c	59.6 ± 11.3	8.3 ± 3.6b	8.8 ± 4.6b
	Shady bottom slope (VS2)	10 ± 0.8a	11.1 ± 1.8a	10.4 ± 1.7a	10.4 ± 1.6a	1.3 ± 0.12a	0.9 ± 0.07b	4.9 ± 1.2a	4.8 ± 1.5c	44.7 ± 13.4c	41.1 ± 11.9b	8.5 ± 4.1b	8.2 ± 6.3b
	Shady middle slope (MS2)	11.9 ± 1.1a	10.1 ± 0.6a	11.9 ± 2.35a	10.4 ± 1.2a	1.1 ± 0.3b	0.9 ± 0.3b	4.7 ± 1.4a	4.1 ± 1.7c	50.1 ± 9.1b	59.1 ± 13.3a	9.4 ± 2.6b	11.2 ± 4.2a
	Shady top slope (RS2)	11.8 ± 1.8a	11.4 ± 0.4a	11.8 ± 1.36a	10.7 ± 1.1a	1.27 ± 0.3a	1.2 ± 0.3a	3.8 ± 1.4b	4.5 ± 1.7c	60.8 ± 8.8a	68.8 ± 13.3a	13.3 ± 1.6a	10.2 ± 4.1a
*C. coggygria* Scop.	Sunny bottom slope (VS1)	12.8 ± 2.5a	12.6 ± 4.9a	11.3 ± 3.9a	9.5 ± 2.1a	0.8 ± 0.2a	0.6 ± 0.2a	2.6 ± 0.39b	2.1 ± 0.7c	34.1 ± 12.5a	32.2 ± 10.4a	14.4 ± 8.1a	12.1 ± 3.9a
	Sunny middle slope (MS1)	11.9 ± 2.6a	12.2 ± 3.6a	9.1 ± 3.2ab	8.2 ± 1.5b	0.7 ± 0.2a	0.6 ± 0.02a	2.8 ± 1.9b	2.8 ± 0.4b	23.7 ± 10.3b	22.1 ± 4c	8.9 ± 3.8c	8.6 ± 2.5b
	Sunny top slope (RS1)	11.9 ± 3.5a	12.1 ± 3.6a	7.5 ± 2.1b	6.9 ± 1.2c	0.8 ± 0.1a	0.6 ± 0.4a	2.5 ± 0.9b	2.5 ± 1.8b	18.2 ± 3.1c	17.2 ± 6.6c	6.4 ± 1.2d	7.9 ± 3.2bc
	Shady bottom slope (VS2)	12.2 ± 1.9a	12.8 ± 2.4a	10.4 ± 1.2a	10.2 ± 2.5a	0.8 ± 0.1a	0.7 ± 0.1a	3.6 ± 0.8a	3.5 ± 0.9a	20.2 ± 3.4c	18.2 ± 6.3c	8.6 ± 2.3c	7.9 ± 1.4bc
	Shady middle slope (MS2)	12.1 ± 1.3a	11.4 ± 4.9a	9.6 ± 0.5ab	8.9 ± 2.2b	0.7 ± 0.2a	0.6 ± 0.1a	3.5 ± 1.6a	2.8 ± 0.1b	28.2 ± 12.9b	25.5 ± 14.8b	13.4 ± 13.1a	8.6 ± 4.1b
	Shady top slope (RS2)	12.0 ± 1.3a	12.2 ± 4.4a	7.7 ± 0.5b	7.9 ± 1.7c	0.7 ± 0.7a	0.5 ± 0.1a	3.6 ± 1.6a	3.6 ± 0.8a	17.7 ± 17.4c	17.6 ± 14.1c	11.1 ± 3.9b	6.6 ± 1.1c

MBC: microbial biomass C; MBN: microbial biomass N; MBP: microbial biomass P

There were significant differences between different slope positions of the same tree species and are represented by different lowercase letters (p < 0.05)

**Table 3:** Effect of tree species, topography and their combined effect on the C, N and P content in soil and microbial biomass and their ecological stoichiometry

Soil layer	Component	Tree species		Slope aspect		Slope position		Vegetation * aspect		Vegetation * slope position		P × A		V × P×A	
		F	P	F	P	F	P	F	P	F	P	F	P	F	P
0–10	C	78.983	0.000	22.67	0.000	9.365	0.000	13.275	0.000	17.741	0.000	0.511	0.927	0.885	0.898
	N	66.782	0.000	14.249	0.001	5.365	0.011	7.726	0.002	8.879	0.004	0.509	0.943	0.786	0.789
	P	25.556	0.000	9.397	0.008	0.128	0.99	2.231	0.227	2.289	0.183	1.211	0.479	0.595	0.911
	C:N	9.98	0.003	1.399	0.408	1.781	0.324	1.402	0.414	2.521	0.144	2.443	0.17	0.524	0.978
	C:P	88.235	0.000	17.848	0.000	15.523	0.000	10.203	0.000	17.909	0.000	0.95	0.707	1.211	0.804
	N:P	75.354	0.000	10.502	0.003	6.923	0.012	7.604	0.01	8.379	0.007	0.745	0.827	1.23	0.794
	MBC	55.698	0.000	14.239	0.001	15.115	0.000	9.345	0.003	11.204	0.000	0.523	0.997	0.813	0.734
	MBN	29.358	0.000	8.978	0.014	5.324	0.03	3.88	0.044	5.244	0.018	0.503	0.979	0.58	0.944
	MBP	56.234	0.000	18.209	0.000	4.173	0.041	13.91	0.000	2.31	0.087	0.649	0.71	0.68	0.839
	MBC:MBN	5.632	0.003	1.227	0.398	0.078	0.785	1.651	0.328	0.279	0.928	0.705	0.874	1.595	0.344
	MBC:MBP	3.139	0.018	0.514	0.908	1.114	0.231	8.309	0.007	0.609	0.899	0.581	0.809	3.703	0.07
	MBN:MBP	2.472	0.097	0.521	0.982	0.423	0.685	1.68	0.413	0.609	0.936	0.551	0.827	2.844	0.108
10–20	C	77.862	0.000	12.923	0.009	44.521	0.000	4.204	0.038	28.229	0.000	0.586	0.773	1.823	0.282
	N	68.256	0.000	8.27	0.023	33.657	0.000	2.775	0.117	18.42	0.000	1.784	0.283	4.886	0.018*
	P	9.9654	0.000	1.918	0.24	0.321	0.985	1.389	0.419	1.576	0.377	0.579	0.787	0.629	0.883
	C:N	4.258	0.017	0.783	0.797	0.072	0.82	0.8	0.743	0.91	0.889	0.942	0.71	2.715	0.121
	C:P	87.356	0.000	4.548	0.04	25.525	0.000	2.651	0.128	19.33	0.000	0.526	0.878	1.403	0.413
	N:P	69.354	0.000	3.429	0.094	18.365	0.000	1.88	0.288	12.602	0.000	0.905	0.729	3.981	0.04
	MBC	54.356	0.000	2.277	0.218	21.328	0.000	0.839	0.718	17.736	0.000	0.576	0.999	0.579	0.932
	MBN	34.526	0.000	0.749	0.82	6.68	0.023	0.703	0.819	4.31	0.037	0.551	0.827	0.85	0.709
	MBP	33.354	0.000	2.573	0.179	3.356	0.124	2.419	0.179	1.389	0.708	0.624	0.728	0.631	0.879
	MBC:MBN	7.824	0.007	1.509	0.321	0.965	0.651	1.34	0.44	1.581	0.612	0.73	0.837	1.731	0.302
	MBC:MBP	4.412	0.042	1.307	0.441	0.058	0.985	4.21	0.032	1.398	0.717	0.615	0.738	0.716	0.808
	MBN:MBP	1.238	0.174	1.333	0.439	0.005	0.985	5.285	0.013	1.286	0.483	0.505	0.98	0.733	0.797

The soil microbial biomass C:N ratios of *Q. variabilis* and *L. principis*-*ruprechtii* were relatively high on the sunny slope. The soil microbial biomass C:N ratios of *C. coggygria* Scop. was highest in the shady top slope, and there was a significant difference in the soil microbial biomass C:N ratios between different slope positions. The soil microbial biomass C:P ratios of *Q. variabilis* and *C. coggygria* Scop. soil were highest in the sunny bottom slope, making them significantly different from those of the other slope positions. The soil microbial biomass C:P ratio of *L. principis*-*ruprechtii* was the highest in the shady top slope, and it was significantly different from that of the other slope positions. The variation trend of the soil microbial biomass N:P was consistent with that of the soil microbial biomass C:P.

### Correlations among soil C, N, P, MBC, MBN, MBP, and their ecological stoichiometry

**Fig. 5:**
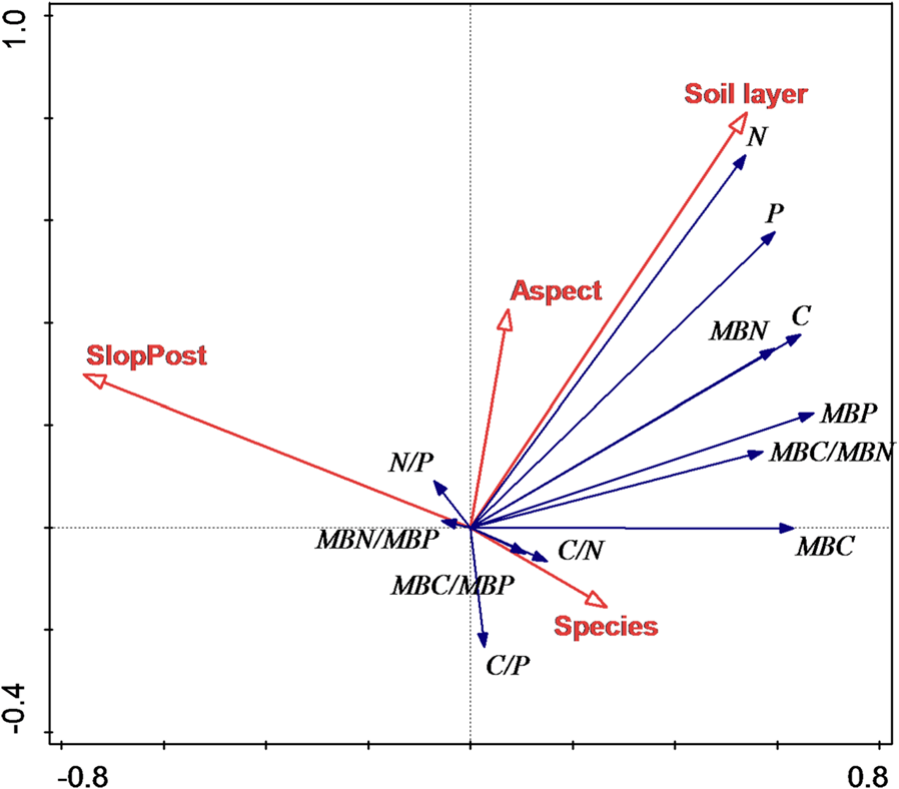
Effects of environmental factors (topography, tree species and soil layers) on soil and microbial biomass stoichiometry according to redundancy analysis

To explore the separate contributions of the environmental factors to soil and microbial biomass stoichiometry, RDA was performed, and the results are shown in Fig. 5. Tree species exhibited a significant impact on soil and microbial biomass stoichiometry (P = 0.002), with a contribution rate of 57.1%. The contribution rate of topographic factors, including slope aspect and position, was only 36.2%. Soil layers did not show a noticeable impact on C:N:P stoichiometry, whose contribution rate was only 6.7%.

As shown in Table 4, there was a strongly significant correlation between soil C, N and P and between soil MBC, MBN and MBP. Additionally, there was a strongly significant correlation between soil C, N and P and soil MBC, MBN and MBP. No significant correlation was found between soil C, N, P and MBC, MBN, and MBP with soil C:N. However, there was a strongly significant correlation between soil C:N and N:P. Additionally, soil N, P and MBC and N:P influenced soil MBC:MBN. Basically, there was no significant correlation between soil C, N, and P and soil MBC, MBN and MBP with MBC:MBP and MBN:MBP. In addition, no significant correlation was found between soil N:P and soil microbial biomass N:P.

**Table 4:** Correlation coefficient matrix of soil C, N, and P and soil MBC, MBN, and MBP contents and their ecological stoichiometry

	N	P	MBC	MBN	MBP	C::N	C:P	N::P	MBC:MBN	MBC::MBP	MBN::MBP
C	0.908**	0.920**	0.925**	0.925**	0.919**	0.289	0.929**	0.978**	0.385	− 0.020	− 0.276
N		0.912**	0.957**	0.936**	0.914**	0.251	0.948**	0.978**	0.368*	0.051	− 0.287
P			0.866**	0.836**	0.828**	0.124	0.889**	0.932**	0.496**	− 0.052	− 0.323
MBC				0.958**	0.942**	0.280	0.934**	0.954**	0.398*	0.056	− 0.234
MBN					0.953**	0.233	0.934**	0.924**	0.276	− 0.032	− 0.252
MBP						0.242	0.878**	0.898**	0.382	− 0.124	− 0.472*
C:N							0.320	0.198	− 0.269	− 0.456**	− 0.153
C:P								0.958^*^	0.325	− 0.010	− 0.234
N:P									0.410*	0.043	− 0.275
MBC:MBN								^*^		0.423	− 0.268
MBC:MBP											0.557^**^

*p < 0.05; **p < 0.01

### Correlation between soil C, N, and P and soil MBC, MBN, and MBP contents and their ecological stoichiometry with environmental factors

Pearson correlation analysis also showed that at 0–10 cm, the soil C, N, and P contents and their ecological stoichiometry were closely related to tree species. The environmental factor analysis showed that the tree species factor could account for 80.5% of the variation, and the slope aspect and position and other topographic factors could account for 8.8% of the variation. The results obtained were basically consistent at both depths of 10–20 and 0–10 cm. Tree species was also the most important influencing factor, being responsible for more than 70% of the variation.

The results of SMA analysis of soil MBC, MBN, and MBP and soil C, N, and P indicated that the slopes between MBC and MBN, between MBC and MBP, and between MBN and MBP were all approximately equal to 1, showing a well-constrained proportion and indicating an isometric relation between soil MBC, MBN and MBP. The slopes of SOC, soil MBC, soil TP and soil MBP were significantly greater than 1 (Table 5). The relationships between soil C, N and P and soil MBC, MBN and MBP presented the characteristics of a nonisometric model, revealing a strong dependence of soil microbes on soil nutrient content.

**Table 5:** SMA analysis of soil C, N and P and soil MBC, MBN and MBP contents

*x*	*y*	Slope	*r*^2^	*P*
SOC	TN	*0.97*	0.798	< 0.001
SOC	TP	0.885	0.368	< 0.001
TN	TP	0.834	0.485	< 0.001
MBC	MBN	*0.968*	0.842	< 0.001
MBC	MBP	*0.924*	0.878	< 0.001
MBN	MBP	*0.998*	0.493	< 0.001
MBC	MBN	1.168	0.345	< 0.001
SOC	MBP	1.212	0.189	< 0.001
SOC	MBP	1.536	0.095	< 0.001
SOC	MBC	1.321	0.158	< 0.001
TN	MBN	1.213	0.201	< 0.001
TP	MBP	1.652	0.125	< 0.001

The slopes close to 1 are shown in italicface, indicating an equidistant or linear relationship between nutrients

## Discussion

Different tree species have different litter yields and physicochemical properties, and the amount of nutrient content returned to soil may also vary [40]. The litter layer covering the soil surface can regulate microclimate conditions, such as water moisture and temperature, and impact the nutrient cycling rate of ecosystems [15, 41, 42]. In this study, tree species had a strongly significant impact on the soil C, N and P and soil MBC, MBN and MBP contents (Fig. 5). There is always a thick layer of litter over the soil surface of *C. coggygria* Scop., making it conducive to accumulating precipitation and reducing water evaporation from the soil mass; the litter layer of *L. principis*-*ruprechtii* is relatively thin but difficult to decompose; the differences in nutrient and moisture contents resulted in the difference in soil microbial biomass [30]. For example, Devi and Yadava [4] showed that soil moisture content had a strong impact on soil microbial biomass, and tree species significantly affected soil and soil MBC, MBN, and MBP contents. Patel et al. [43] also thought that changes in land use significantly affected soil MBC, MBN and MBP contents. Zhu et al. [14] also concluded that different vegetation restorations on the Loess Plateau had significant impacts on soil C and N and on soil MBC and MBN.

Slope aspect, position and other topographic factors affect the transport, accumulation and distribution of nutrients. In the Funiu Mountain area, topographic factors affect soil microclimate conditions. Compared with the sunny slope, the shady slope has a relatively low rate of water evaporation and has relatively good moisture conditions, which in turn affect the community composition and species of plants [43–46]. In this study, at 0-10 cm, both slope aspect and position significantly influenced MBC, MBN and MBP contents. Soil MBC, MBN and other content indicators, which are sensitive to environmental change, can be used as indicators of soil quality change [3]. The water content is relatively deficient in this study area. Soil MBC, MBN, and MBP are more sensitive to environmental change than are soil C, N, and P. The better water content condition in the shady slope is favorable to microbial growth and reproduction, facilitating nutrient transformation. The significant correlation between the soil C, N and P contents and the soil MBC, MBN, and MBP contents revealed that shady slopes, compared with sunny slopes, have higher soil C, N and P contents and soil MBC, MBN and MBP contents. The better water and nutrient conditions in the shady slope facilitate the growth of tree species. Moreover, the shady slope has a thicker litter layer (Table 1), which is particularly obvious for *C. coggygria* Scop. (Figure 1). At 10–20 cm, the impact of slope position on the soil C and N contents reached a very significant level, while the impact of slope position on the soil C and N contents reached a significant level. Slope position had a significant impact on soil MBC and MBN, while slope position did not. At 0–10 cm, aspect was a more important influencing factor. In contrast, at 10–20 cm, slope position was a more important factor in terms of its influence on the soil C, N and P and soil MBC, MBN and MBP contents. Zhu et al. [14] found that the effect of different tree species on soil organic C and N and soil MBC and MBN was greater than the effect of topographic factors (such as slope position) in the Loess Plateau, consistent with the results of this study. At the same time, the SMA analysis results indicate that the soil C, N and P contents and soil MBC, MBN, and MBP contents show a nonisometric relationship (Table 5), the slope between soil MBC and soil C is significantly greater than 1, and the slope between soil MBP and soil P content is even greater. A low availability of soil P and the different activation efficiency of soil P by soil microbes in different locations contribute to this result, indicating that soil microbes are strongly reliant on soil nutrient content, especially soil P, which may be attributed to the fact that P comes only from soil.

The change in plant species is a main factor affecting the soil C:N ratio. The difference in nutrient proportion and the nutrient cycling mechanism within plants impacts litter, which in turn affects soil C:N. At the same time, soil C:N usually reflects the decomposition rate of soil organic matter, and a low C:N ratio represents rapid decomposition of organic matter. In estimating the global soil carbon stock, the soil C:N ratio (10:1) is generally regarded as a constant; generally, the correlation analysis of soil C, N, and P and soil MBC, MBN and MBP with soil C:N suggests that there is no correlation between them, reflecting the stability of soil C:N on a microtopography scale [47, 48]. The results of this study were consistent with those reported in the literature [47, 48]: Slope aspect and position impact only C:P and N:P and have no impact on C:N. Soil ecological stoichiometry also vary according to soil and other microtopography factors [13].

Tree species also have a significant impact on soil MBC:MBN and MBC:MBP (Fig. 5), and their impact is greater at 0–10 cm than at 10–20 cm, which is consistent with the conclusions reached by Cleveland and Liptzin [6]. Tree species, as a potential factor, influences soil microbial ecological stoichiometric ratios. Different tree species may lead to changes in litter quantity and quality or changes in the main composition of the microbial community. Heuck et al. [1] discovered that the ecological stoichiometry of soil microbial biomass varied in soil where P was deficient, which might be related to the change in the composition of the soil microbial community. Different microbial communities with different vegetation type conditions may cause changes in soil MBC:MBN and MBC:MBP. Tischer et al. [49] found that soil and microbial biomass stoichiometry varied according to language use and soil depths and that the soil C:N:P ratio noticeably decreased after forests were interference. However, our study found that tree species had an impact on soil MBC:MBN and MBC:MBP but no impact on soil MBN:MBP. Different from the impact of tree species on soil MBC:MBN and MBC:MBP, slope aspect and position had no impact on the ecological stoichiometry of soil microbial biomass, reflecting its stability on a microtopography scale. Only tree species and slope direction had a combined effect on the ecological stoichiometry of soil microbial biomass. These findings may be related to the unique climate conditions in the Huaihe River basin. Tree species, as the most important influencing factor, impacted soil MBC:MBN and MBC:MBP.

The homeostasis of organism stoichiometry, as a central concept in ecological stoichiometry, is used to reflect the maintenance and circulation of nutrients on scales ranging from subcellular to ecosystem. The homeostasis of ecological chemistry is defined as the extent to which an organism maintains the stability of its own chemical composition when the chemical composition and availability of external resources vary [5]. The slope formed by the element content of organisms and the element content of resources after logarithmic transformation is called homeostasis. Sterner and Elser [5] defined a slope less than 1 as homeostasis, while McGroddy et al. [50] defined a slope equal to 1 as homeostasis. Cleveland and Liptzin [6] conducted an SMA analysis of forest soil and grassland soil microbial biomass and found that the slope of soil microbial biomass C-N, C-P and N-P was approximately 1 or less than 1 and that soil microbial biomass C was significantly related to N and P, indicating that soil microbes had homeostasis.

In this study, Pearson correlation analysis showed significant relationships among MBC, MBN and MBP. In addition, the SMA analysis showed that the slopes formed between soil MBC, MBN and MBP were smaller than—yet close to—1, with a well-constrained ratio. These findings were basically consistent with those reported by Cleveland and Liptzin [6], which indicated that soil microbes had homeostasis. Fanin et al. [51] studied the homeostasis of microbes in the decomposition process of plant litter and found that the slope formed between microbial N and P was significantly greater than 1, showing that they did not possess homeostasis. This result is not consistent with the results of this study and those of Cleveland and Liptzin [6], and this difference may be related to the environmental conditions in which microbes live. There is a relatively stable environment in the soil. When microbes are deficient in elements, they can be obtained from the soil. Unlike soil, a stable environmental condition is absent in the litter. The lack of elements necessary for the formation of microbes during leaf decomposition may cause microbes to rely heavily on N or P and result in a change in homeostasis.

Plant N:P usually varies with changes in tree species or vegetation [6, 51], mainly due to the biogeographical gradient difference in soil matrix age. In this study, the change in tree species did not have a significant impact on soil microbial biomass N:P. There was no relationship between the supply ratio of soil MBN:MBP and soil N:P, which was consistent with the results of Cleveland and Liptzin [6], indicating that soil microbial biomass N:P conforms to the “Redfield” ratio and that homeostasis controls the ratio of elements. In this study, slope aspect and position had little impact on the ecological stoichiometry of soil microbial biomass (Fig. 5). Correlations showed that the C, N and P contents of soil and microbial biomass and their ecological stoichiometry had no correlation with MBN:MBP. From the perspective of environmental factors, soil microbes exhibited homeostasis. N and P are generally deemed the most important limiting elements to plants in terrestrial ecosystems. The critical ratio of N:P in plant leaves is thought to be an indicator that can be used to assess the nutrient supply of the environment to plant growth [11, 37, 51–54]. When N:P is smaller than 14, N is limited; when N:P is greater than 16, P is limited; when N:P is between 14 and 16, the ecosystem is limited by both N and P or no limitations exist due to a sufficient nutrient supply. In areas with complex topography, the N:P ratio of plant leaves is usually used to indicate nutrient limitation in soil.

In tropical ecosystems, soil microbial biomass N:P could also be used to reflect nutrient limitation in soil, and a high soil microbial biomass N:P indicates the presence of P limitation [6]. The N:P ratio of plant leaves has a very wide range [6]. The range of measured leaf ratios included the above N:P breakpoint value [55]; thus, it was not possible to evaluate the N and P limitations at this site. However, the N:P ratio of soil microbial biomass was limited, and its range was much narrower than that of plant leaves. Therefore, the N:P ratio could better reflect the nutrient limitation of soil. Ren et al. [10] found that a high N:P ratio in soil microbes in the vegetation restoration area of the Loess Plateau demonstrated that plant growth was limited by P. In this study, the average soil microbial biomass N:P of tree species was 10.9 (equivalent to the atomic ratio of 24.1), which suggested that there was a relative lack of N in this area and that tree species may be mainly limited by N. This result was consistent with the conclusion reached by Bai et al. [56], i.e., forest region was restricted by N. This study also found that soil microbial biomass N:P was not sensitive to tree species variation, indicating that the homeostasis of soil microbial biomass N:P was better than that of plant leaves [6], which may be used to reflect the nutrient limitation of ecosystems. In this study area, the leaf N:P ratio of tree species could be combined with the soil microbial biomass N:P ratio to evaluate the nutrient limitations of ecosystems more accurately, providing a basis for ecosystem restoration and management.

Tree species and topography have significant impacts on the ecological stoichiometry of soil and soil microbial biomass, and soil microbes have homeostasis. Our research results are limited to only a small basin in Funiu Mountain, China. Only by a statistical analysis of a large set of sample data covering different climate zones and land uses across the world is it possible to reveal the impact mechanism of tree species and topography on the ecological stoichiometry of the soil and soil microbial biomass.

In conclusion, compared with topographic factors, tree species have a greater impact on soil nutrient content and microbial biomass. Under the same tree species conditions, aspect is the most important factor influencing soil nutrient content and soil microbial biomass at a soil depth of 0–10 cm, whereas slope position has a greater impact at 10-20 cm. There was a very significant correlation between the soil C, N and P contents and the soil MBC, MBN and MBP contents. SMA analysis also revealed that soil microbes heavily rely on soil nutrients, especially soil P, reflecting a close relationship between soil microbes and soil nutrient cycling. However, no correlation was found between the stoichiometric ratios of soil microbial biomass, soil C, N and P contents and their stoichiometric ratios with the slope aspect and position and other topographic factors. According to the SMA analysis of the soil MBC, MBN, and MBP, the soil microbial biomass in the investigated area has a well-constrained ratio, which suggests homeostasis of the soil microbes. The insensitivity of MBN:MBP to tree species and topographic variations and the homeostasis of the microbes may enable the ratio to be used to assess the nutrient limitations of ecosystems.

## Data Availability

The datasets analyzed during the current study are available from the corresponding author upon reasonable request.

## Abbreviations

CFE: Chloroform fumigation extraction
GLM: General linear model
TN: Total nitrogen
TP: Total phosphorus
MBP: Microbial biomass phosphorus
